# Nitric oxide augments signaling for contraction in hypoxic pulmonary arterial smooth muscle—Implications for hypoxic pulmonary hypertension

**DOI:** 10.3389/fphys.2023.1144574

**Published:** 2023-03-29

**Authors:** Martha Hinton, James A. Thliveris, Grant M. Hatch, Shyamala Dakshinamurti

**Affiliations:** ^1^ Biology of Breathing Group, Children’s Hospital Research Institute of Manitoba, Winnipeg, MB, Canada; ^2^ Department of Physiology and Pathophysiology, University of Manitoba, Winnipeg, MB, Canada; ^3^ Department of Human Anatomy and Cell Science, University of Manitoba, Winnipeg, MB, Canada; ^4^ Department of Pharmacology and Therapeutics, University of Manitoba, Winnipeg, MB, Canada; ^5^ Department of Pediatrics, Section of Neonatology, Health Sciences Centre, Winnipeg, MB, Canada

**Keywords:** persistent pulmonary hypertension of the newborn, nitric oxide, hypoxia, smooth muscle, thromboxane, calcium, adenylyl cyclase

## Abstract

**Introduction:** Hypoxic persistent pulmonary hypertension in the newborn (PPHN) is usually treated with oxygen and inhaled nitric oxide (NO), both pulmonary arterial relaxants. But treatment failure with NO occurs in 25% of cases. We previously demonstrated that 72 h exposure to hypoxia, modeling PPHN, sensitized pulmonary artery smooth muscle cells (PASMC) to the contractile agonist thromboxane and inhibited relaxant adenylyl cyclase (AC) activity.

**Methods:** In this study, we examined the effects of sodium nitroprusside (SNP), as NO donor, on the thromboxane-mediated contraction and NO-independent relaxation pathways and on reactive oxygen species (ROS) accumulation in PASMC. In addition, we examined the effect of the peroxynitrite scavenger 5,10,15,20-Tetrakis (4-sulfonatophenyl)porphyrinato Iron (III) (FeTPPS) on these processes.

**Results:** Exposure of PASMC to 72 h hypoxia increased total intracellular ROS compared to normoxic control cells and this was mitigated by treatment of cells with either SNP or FeTPPS. Total protein nitrosylation was increased in hypoxic PASMC compared to controls. Both normoxic and hypoxic cells treated with SNP exhibited increased total protein nitrosylation and intracellular nitrite; this was reduced by treatment with FeTPPS. While cell viability and mitochondrial number were unchanged by hypoxia, mitochondrial activity was decreased compared to controls; addition of FeTPPS did not alter this. Basal and maximal mitochondrial metabolism and ATP turnover were reduced in hypoxic PASMC compared to controls. Hypoxic PASMC had higher basal Ca2+, and a heightened peak Ca2+ response to thromboxane challenge compared to controls. Addition of SNP further elevated the peak Ca2+ response, while addition of FeTPPS brought peak Ca2+ response down to control levels. AC mediated relaxation was impaired in hypoxic PASMC compared to controls but was normalized following treatment with FeTPPS. Addition of SNP inhibited adenylyl cyclase activity in both normoxic and hypoxic PASMC. Moreover, addition of the Ca2+ chelator BAPTA improved AC activity, but the effect was minimal.

**Discussion:** We conclude that NO independently augments contraction and inhibits relaxation pathways in hypoxic PASMC, in part by a mechanism involving nitrogen radical formation and protein nitrosylation. These observations may partially explain impaired effectiveness of NO when treating hypoxic pulmonary hypertension.

## Introduction

Persistent pulmonary hypertension of the newborn (PPHN) represents a catastrophic failure of the natural course of pulmonary vascular relaxation after birth (Dakshinamurti, 2005). It complicates 0.2%–0.6% of births and 10% of NICU admissions, with 1,000 deaths annually in the United States (Farrow et al., 2005; Bhutani, 2008; Steurer et al., 2017). The pathophysiology of endothelial and smooth muscle dysfunction in PPHN is multifactorial; 40% of cases result from hypoxia, ventilation/perfusion mismatch or meconium aspiration, and 25% from inflammation or sepsis. Pulmonary hypertension also complicates other neonatal conditions such as chronic lung disease or congenital diaphragmatic hernia. Despite aggressive vasodilation with nitric oxide (NO), death ranges 10%–25% (Clark et al., 2000; Finer and Barrington, 2006; Barrington et al., 2017). Treatment failure with NO, or an initial response followed by development of NO resistance, occurs in about 25% of cases (Goldman et al., 1996; Pedersen et al., 2018).

Inhaled NO is a mainstay of PPHN treatment, especially in the hypoxemic patient. But complications of NO can include protein nitrosylation, a reversible post-translational modification where NO covalently attaches to the thiol group on a cysteine (sNO) (Gould et al., 2013; Qin et al., 2013). The ratio of O_2_- to NO determines the fate of nitrogen radicals arising from NO or from peroxynitrite (ONOO-) (Espey et al., 2002). Nitrosylation due to sNO formed from ONOO- is favored when O_2_- is increased relative to NO (Espey et al., 2002); while recombinant superoxide dismutase (SOD) improves NO responsiveness of pulmonary artery (Steinhorn et al., 2001; Lakshminrusimha et al., 2006). We previously demonstrated that 72 h exposure to hypoxia, modeling PPHN *in vivo* or *in vitro*, resulted in hyper-responsiveness of pulmonary artery smooth muscle cells (PASMC) to the contractile agonist thromboxane (Hinton et al., 2006; Santhosh et al., 2014). In addition, exposure of PASMC to hypoxia nitrated and inhibited the antioxidant enzyme superoxide dismutase (SOD2) resulting in superoxide (O_2_-) accumulation (Gong et al., 2010). Moreover, exposure of PASMC to hypoxia was shown to nitrosylate and inhibit relaxant adenylyl cyclase (AC) activity (Sikarwar et al., 2018). Thus, during treatment with NO hypoxic pulmonary artery PASMC may be rendered more sensitive to contractile signaling.

Given these potential interactions, we examined the additive effects of NO (as sodium nitroprusside, SNP) on thromboxane-mediated contraction and on NO-independent relaxation pathways in hypoxic PASMC, and on accumulation of reactive oxygen species (ROS) and reactive nitrogen species (RNS). In addition, we examined the effect of the peroxynitrite scavenger FeTPPS on these processes. We hypothesized that hypoxia in PASMC induces mitochondrial changes rendering them prone to ROS and RNS generation, and that the addition of exogenous NO sensitizes the contractile apparatus while inhibiting relaxation.

## Methods

*Primary cell culture model:* All experiments were carried out in accordance with the guidelines of the Canadian Council on Animal Care and approved by the institutional review board. Newborn piglets from a pathogen-free farm supplier (<24 h old; N = 8; 5 male, 3 female) were euthanized by pentobarbital overdose and exsanguination. Heart and lungs were removed *en bloc* and immediately placed in Krebs-Henseleit buffer containing (in mM): 112.6 NaCl, 25 NaHCO3, 1.38 NaH2PO4, 4.7 kC l, 2.46 MgSO4∙7 H2O, and 5.56 Dextrose; pH 7.4; 4 °C. Pulmonary artery smooth muscle cells (PASMC) were dissociated as previously described (Hinton et al., 2006). Briefly, 3^rd^—6^th^ generation pulmonary arteries were dissected and placed in Ca^2+^-free Krebs-Henseleit buffer. Arteries were then washed in cold HEPES buffered saline solution (HBS; containing in mM:130 NaCl, 5 kC l, 1.2 MgCl_2_, 10 HEPES, and 10 glucose; pH 7.4) supplemented with antibiotic-antimycotic and gentamicin starting with high Ca^2+^ (1.5 mM), followed by low Ca^2+^ (20 μM) and then Ca^2+^-free media over a 1 h period in order to select for monotypic collection of PASMC (Shimoda et al., 2000). Tissue was then minced and incubated in a digestion medium (1750 U/ml type I collagenase, 1 mM dithiotreitol, 2 mg/ml bovine serum albumin (BSA), and 9.5 U/ml papain in Ca^2+^-free HBS) for 15min at 37°C with gentle agitation. Digestion medium was washed away from dispersed PASMC with Ca^2+^-free HBS, which was subsequently replaced with culture medium; Ham’s F-12 + 10% fetal calf serum +1% penicillin +1% streptomycin. After 1 passage, PASMC were grown to confluence, then serum-deprived (Ham’s F-12 + 1% penicillin +1% streptomycin +1% insulin transferrin selenium X) to synchronize in a contractile phenotype (Halayko et al., 1997; Fediuk et al., 2012). In the following 3 days, cells were maintained in serum-free media and split into the following conditions: normoxic (N; 21%O_2_, 5%CO_2_) or hypoxic (H; 10%O_2_, 5% CO_2_) with or without daily addition of peroxynitrite scavenger FeTPPS (5,10,15,20-Tetrakis (4-sulfonatophenyl) porphyrinato Iron III), Chloride; 1 μM) or the NO donor SNP (1 μM). Duration of hypoxia exposure was chosen based on characterization of thromboxane receptor sensitization in myocytes from neonatal animals with PPHN, and replication of same in this cell culture model following 3 days hypoxia exposure (Hinton et al., 2006). Agonist doses were selected from the steep sections of published dose response curves for thromboxane challenge (Hinton et al., 2006; Fediuk et al., 2014; Santhosh et al., 2014), for ATP stimulation of adenylyl cyclase (Sikarwar et al., 2018), and for SNP and FeTPPS (Gong et al., 2010).

*ROS and Superoxide Measurement:* PASMC plated in black-well 96-well plates were washed free of media with PBS. H_2_-DCF-DA (to measure total ROS) or DHE (to measure superoxide) was dissolved immediately prior to use in DMSO, then diluted to 100 μM in Ham’s F-12 and incubated for 30min at 37°C. Extracellular probe was washed away 2x with PBS, and the plate was read at an excitation/emission wavelength of 485/520nm, or 500/620 nm for DCF and DHE, respectively.

*Total Nitrosylation:* PASMC were fixed and permeabilized with 4% paraformaldehyde and 0.1% Triton-X. Nitrosylation of proteins was measured using a biotin switch method (*S*-nitrosylated protein kit, Cayman Chemical Inc., Ann Arbor MI) using a fluorescent plate reader per the manufacturer’s instructions.

*Nitrate Measurement:* Nitrate levels were determined by the fluorometric Nitrate/Nitrite Assay Kit (Cayman Chemical) per the manufacturer’s instructions using 20ul of conditioned media.

*Cell Viability:* PASMC seeded in black-well 96-well plates were washed free of media with Calcein AM DW Buffer and then loaded with 1 μM Calcein AM for 30min at 37°C as per the instructions in the Calcein AM Cell Viability Kit (Trevigen Inc., Minneapolis, MN). Plates were read at excitation and emission wavelengths of 490 and 520nm, respectively.

*Metabolic Activity:* PASMC seeded in 96-well plates were maintained in 100 μl total volume. MTT reagent ((3-(4,5-Dimethylthiazol-2-yl)-2,5-diphenyltetrazolium bromide) was added to wells for a final concentration of 0.5 mg/ml and incubated at 37°C for at least 2 h. All liquid was then aspirated, formazan was solubilized in 100μl/well DMSO and absorbance was read at 570 nm.

*Electron Microscopy:* PASMC were fixed in 3% glutaraldehyde in 0.1 M phosphate buffer followed by fixation in 1% osmium tetroxide in 0.1 M phosphate buffer. Samples were processed and embedded in Epon 812 using standard procedures. Thin sections were stained with uranyl acetate and lead citrate, viewed without foreknowledge of their source and photographed on a Philips CM-10 electron microscope. Morphometric analysis was performed using the ZIDAS system (Carl Zeiss Inc., Maple Grove MN). This system determined numbers of mitochondria by point count and distance between mitochondria and cell periphery.

*Mitochondrial Analysis:* Oxygen consumption rate (OCR) was measured in PASMC using a Seahorse Bioscience Extracellular Flux Analyzer (model XF24, Agilent Technologies). Sequential addition of 10uM oligomycin (complex V inhibitor), FCCP (carbonyl cyanide-trifluoromethoxy phenylhydrazone; an uncoupler), 5 µM antimycin A (mitochondrial respiration inhibitor) and 5 µM rotenone (complex I inhibitor) allowed measurement of non-mitochondrial respiration, basal and maximum mitochondrial respiration, ATP production and proton leak measurements, as per the manufacturer’s instructions.

*Western blot:* PASMC were lysed in RIPA buffer; lysates containing equal protein content were loaded onto gels and separated by SDS-PAGE. Proteins were transferred to nitrocellulose membranes and probed with epitope-specific antibodies (rabbit-anti-cleaved caspase-3 (Cell Signaling Technology), rabbit-anti-phospholipase C beta (Novus Biologicals), mouse-anti-smooth muscle α-actin (Sigma Aldrich), rabbit-anti-desmin (Sigma Aldrich), rabbit-anti-adenylyl cyclase 6 (Abcam) or mouse-anti-β-actin (Sigma Aldrich)), visualized by enhanced chemiluminescence and captured using a chemidoc (Biorad) and ImageJ Software.

*Calcium Imaging:* Media was washed away twice from PASMC with HBSS + Ca^2+^+Mg^2+^ +0.1%BSA (HBSS+++). Cells were loaded with 5 μM fura-2AM in HBSS+++ with 1 μg/ml pluronic acid for 1 h at 37°C in a humidified chamber. Extracellular fura-2AM was then removed by washing 4x with HBSS+++ with gentle agitation. De-esterification was allowed for at least 30min. Recordings were obtained at ×20 magnification on an inverted Olympus microscope at 340nm and 380 nm excitation and 510 nm emission wavelengths and captured by NIS- elements software. For each recording, a stable baseline was observed followed by a Ca^2+^ response to 1 μM U46619 (a thromboxane mimetic) and a return to baseline. Background was subtracted from individual files using a cell-free area. Up to 8 identically sized areas per file, containing 3–5 cells and no background, were used to determine basal and peak Ca^2+^. Each run contained all treatment groups, studied in cells obtained from the same culture of PASMC, studied contemporaneously using identical microscope settings, to limit within-group variation; results were normalized against a normoxic treatment-naïve standard for each day of experimentation. The 340/380 nm ratios were converted to [Ca^2+^]i using the Grynkiewiez calculation and a calcium calibration curve derived using the same microscope settings (Grynkiewicz et al., 1985).

*Mitochondrial Membrane Potential:* PASMC grown in black-well 96-well plates were washed free of media with HEPES buffer (containing in mM; 40 HEPES, 25 glucose, 0.1 NaCl; pH 7.4). JC-1 (2.5 μM), a mitochondria-specific cationic dye that differentially fluoresces depending on membrane potential, was loaded into cells for 30min at 37°C. After extracellular JC-1 was washed off, each well was read sequentially over a 90 min period at the excitation/emission wavelength pairs; 490/540 and 540/590. The ratio of 590nm–540 nm fluorescent intensities was used to calculate membrane potential.

*Adenylyl Cyclase (AC) Activity Assay:* Cell lysates were collected and adjusted to 5 µg protein/µl, in 20 mM Tris buffer with protease inhibitors. The activity assay was carried out in 96-well black bottomed plates, each well containing 1 uM ATP in buffer containing 0.25 mM TerbiumIII, 0.05 mM Norfloxacin, 10mM MgCl_2_, 20 µg CaCl_2_, 20 mM Tris-HCl, and 1% BSA, with the reaction commencing by addition of lysate. Fluorescence intensities (time resolved) were acquired by using a BMG FLUOstar OPTIMA microplate reader (Jena, Germany). Excitation/Emission filters were set to 337 and 545 nm, respectively and were performed at 37°C. Data is represented as Δfluorescence/min/mg protein.

*Statistical Analysis:* Data was routinely tested for normality by GraphPad (Prism) software using D'Agostino-Pearson omnibus-K2 normality test; and analyzed by *t*-test or one-way ANOVA with *post hoc* Tukey test for multiple comparisons where appropriate; *p* < 0.05 was considered statistically significant. All independently measured data points (n) were collected from at least 3 biological replicates from independent primary cultures (N) and are presented as mean ± SEM.

## Results

### ROS and RNS

**FIGURE 1:**
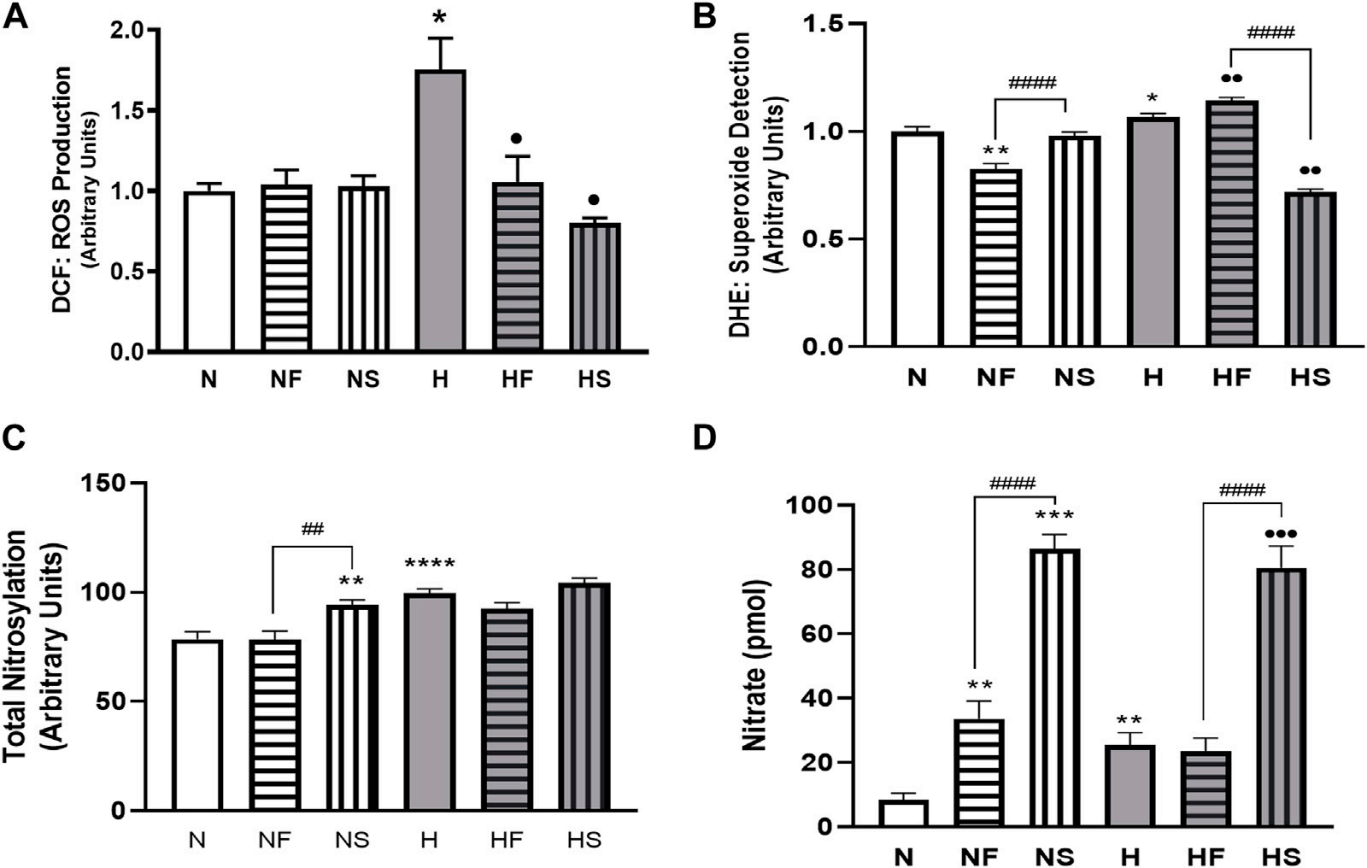
Effects of hypoxia or nitrosylating stress on reactive oxygen and nitrogen species. PASMC were exposed to 72 h of normoxia (N; 21%O2) or hypoxia (H; 10%O2) with or without daily addition of 1uM FeTPPS (F) or 1uM SNP (S). Reactive O2 species **(A)**; DCF; N = 3, n = 10), superoxide **(B)**; DHE; N = 4, n = 8–16), total nitrosylation **(C)**; biotin switch assay; N = 3, n = 6–8). Conditioned media was collected and analyzed for nitrate concentration **(D)**; N = 4, n = 8). *p < 0.05, **p < 0.01, ***p < 0.001, ****p < 0.0001 compared to N. ••*p* < 0.01, •••*p* < 0.001 compared to H. # 0 < 0.05, ##*p* < 0.01, ####*p* < 0.0001 compared between FeTPPS and SNP treatments within similar O2 environments.

72 h exposure of PASMC to hypoxia increased total intracellular ROS (measured by DCF) compared to normoxic cells (Figure 1A). Addition of the NO donor SNP, or the peroxynitrite scavenger, FeTPPS, did not alter ROS in cells grown in 21% O_2_. However, simultaneous treatment with either SNP or FeTPPS attenuated the hypoxia-induced increase in ROS. Superoxide was increased following exposure of PASMC to hypoxia compared to controls (Figure 1B). Treatment with FeTPPS significantly decreased superoxide in normoxic cells, but increased its level in hypoxic cells. Addition of SNP decreased accumulation of superoxide only in hypoxic PASMC.

Total protein nitrosylation was increased in hypoxic PASMC compared to normoxic controls (Figure 1C). In addition, SNP treatment of normoxic cells resulted in elevated nitrosylation. Peroxynitrite scavenging did not alter total nitrosylation levels and simultaneous addition of SNP to hypoxic PASMC did not produce an additive effect. Both normoxic and hypoxic cells treated with SNP exhibited an increase in intracellular nitrite (Figure 1D). Treatment with FeTPPS or hypoxia alone resulted in a smaller, but significant, increase in nitrate. Hypoxic PASMC treated with FeTPPS did not exhibit significantly elevated nitrate levels compared to controls.

### Mitochondrial Analysis

**FIGURE 2:**
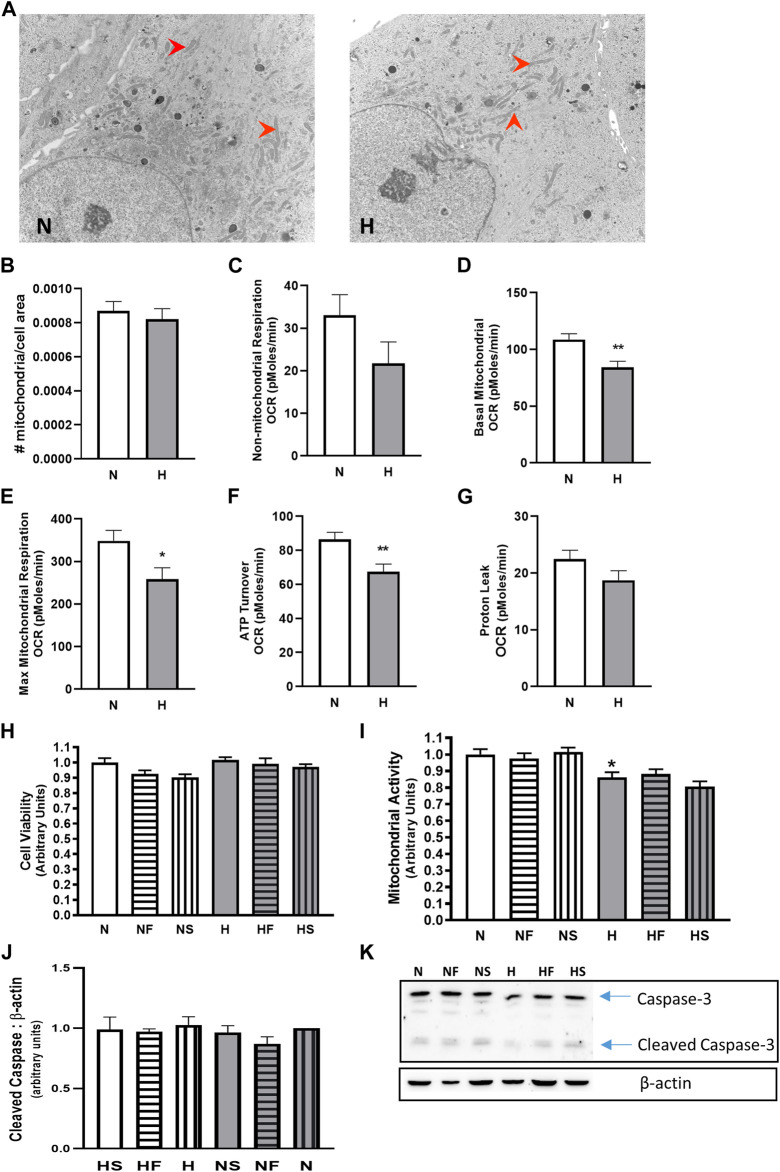
Effect of hypoxia on mitochondria abundance and cellular respiration. PASMC were exposed to 72 h s of normoxia (N; 21%O2) or hypoxia (H; 10%O2) and fixed for EM evaluation of mitochondria (red arrows) density by EM **(A)**, **(B)**; N = 3, n = 22–31). Oxygen consumption was evaluated using a Seahorse analyzer (N = 3, n = 20). Non-mitochondrial **(C)**, basal mitochondrial (**D**) maximal mitochondrial (**E**) respiration were compared, as well as ATP turnover (**F**) and proton leak (**G**). Cell viability **(H)**; calcein assay; N = 5, 6, n = 20–25) and mitochondrial activity assay **(I)**; MTT; N = 3, n = 18) were also measured with and without daily addition of 1uM FeTPPS (F) or 1uM SNP (S). Apoptosis was compared by comparison of cleaved caspase-3 **(J)**; N = 5, n = 5; **(K);** representative blot), *p < 0.05, **p < 0.01.

We examined mitochondrial activity since mitochondria are a large contributor to cellular ROS generation. Apoptosis levels were minimal, as indicated by the low abundance of cleaved caspase-3 (Figures 2J,K), and were unchanged between all treatment groups. While cell viability (Figure 2H) and mitochondrial number (Figures 2A,B) were unchanged by hypoxia, mitochondrial activity, as measured by MTT assay, was reduced in hypoxic PASMC compared to controls (Figure 2I). Addition of FeTPPS restored mitochondrial activity to control values. Basal and maximal mitochondrial respiration and total ATP turnover were decreased following exposure of PASMC to 72 h hypoxia compared to controls (Figures 2D–F). In addition, proton leak (Figure Fig2G) and non-mitochondrial respiration (Figure 2C), while lower, were not significantly altered in hypoxic PASMC compared to controls.

### Contractile pathways

**FIGURE 3:**
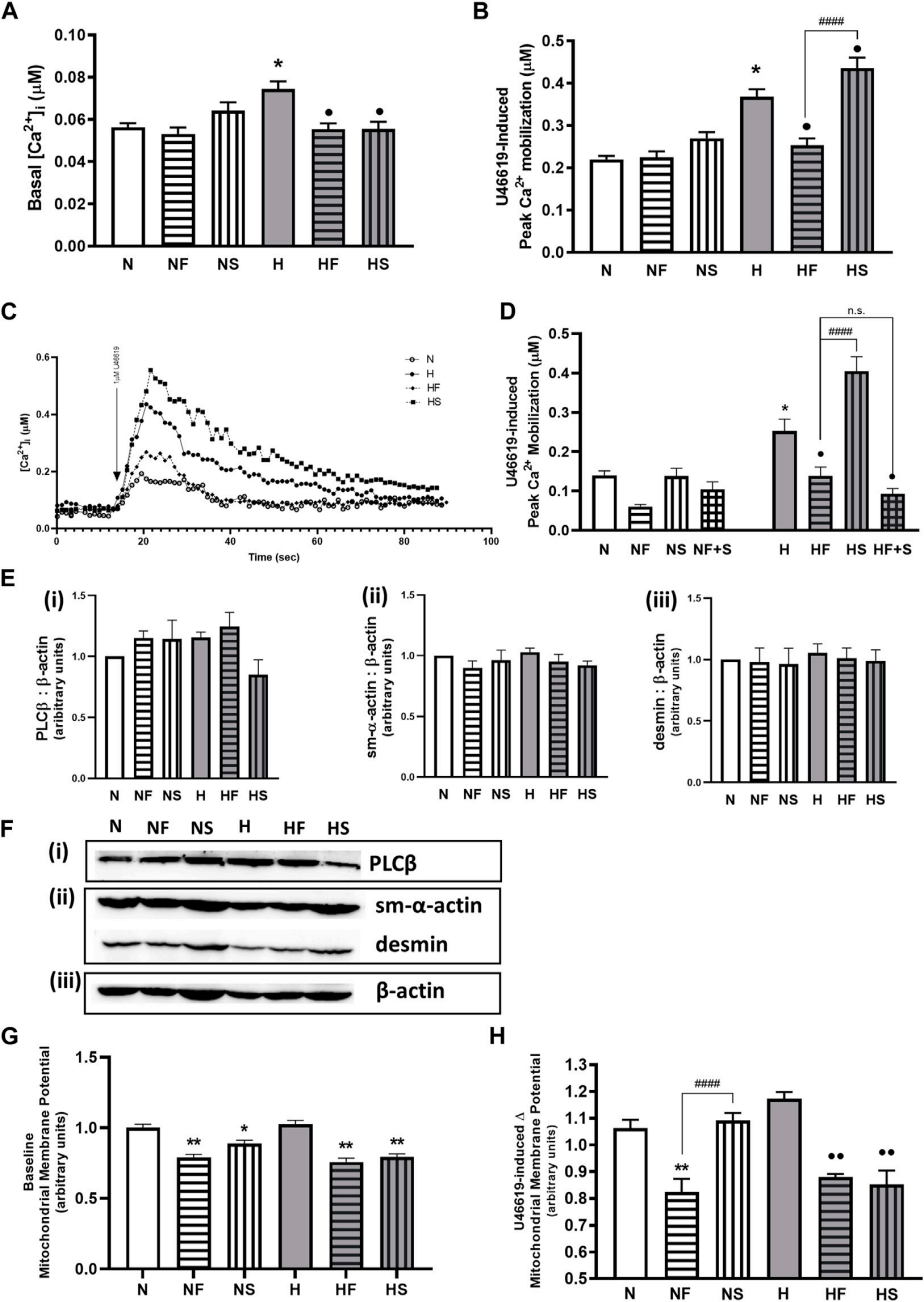
Effect of hypoxia or nitrosylating stress on PASMC contractile mediators. Basal Ca2+ **(A)**; N = 7–10, n = 54–182) and peak Ca2+ mobilization to 1uM U46619 **(B)**; N = 7–19, n = 53–213) in fura-2AM loaded PASMC after exposure to 72 h s of normoxia (N; 21%O2) or hypoxia (H; 10%O2) with or without daily addition of 1uM FeTPPS F) or 1uM SNP (S) were compared **(C)**; representative traces). The effect of daily addition of both 1uM FeTPPS and 1uM SNP (F + S) during the same 72h period on U46619-induced Ca2+ mobilization was also studied **(D)**; N = 3, n = 14–24). Abundance of contractile Ca2+- cascade enzyme, phospholipase C β (PLCβ; **(E(i))**, N = 4; **(F(i))**, representative blot), contractile protein filaments, smooth muscle α-actin (sm-α-actin; **E(ii)**, N = 4; **F(ii)** representative blot) and desmin **(E(iii))**, N = 4; (**F(ii))**, representative blot) were compared and normalized to β-actin **(F(iii))**, representative blots shown; N = 4). Similarly, basal **(G)**; N = 3, 4, n = 14–16 and agonist-stimulated **(H)**; N = 3, n = 8) mitochondrial membrane potential was measured in JC-1 loaded PASMC. *p < 0.05, **p < 0.01 compared to N. •*p* < 0.05, ••*p* < 0.01 compared to H. ####*p* < 0.0001 compared between FeTPPS and SNP treatments within similar O2 environments.

We initially confirmed our previous findings that hypoxic PASMC had higher basal Ca2+, as well as heightened peak Ca2+ response to thromboxane challenge. Addition of FeTPPS during hypoxic exposure to PASMC prevented alterations in resting or peak Ca2+ following thromboxane challenge (Figures 3A–C). Addition of SNP during hypoxic exposure of PASMC further elevated the peak Ca2+ response, but decreased basal Ca2+. Neither FeTPPS nor SNP had any effect on resting or stimulated normoxic PASMC. Simultaneous treatment with FeTPPS and SNP resulted in a significant reduction in peak Ca2+ response to U46619 in hypoxic PASMC (Figure 3D), but had no effect on Ca2+ mobilization in normoxia.

Abundance of phospholipase Cβ (PLCβ, key enzyme in the Ca^2+^ mobilization pathway), is unchanged by hypoxia or following treatment with either FeTPPS or SNP (Figures 3Ei,Fi). Abundance of smooth muscle contractile filament marker proteins α-actin (Figure 3E(ii)) and desmin (Figure 3Eiii) are also unchanged (Figure 3F(ii)).

Basal mitochondrial membrane potential was lowered by both SNP and FeTPPS regardless of oxygen environment (Figure 3G. During stimulation of a Gαq-linked G protein coupled receptor, mitochondrial membrane potential was decreased in the presence of FeTPPS (Figure 3H). Addition of SNP lowered stimulated hypoxic PASMC mitochondrial membrane potential. Exposure to hypoxia alone did not elevate mitochondrial potential above baseline in normoxic PASMC.

### Relaxation pathways

**FIGURE 4:**
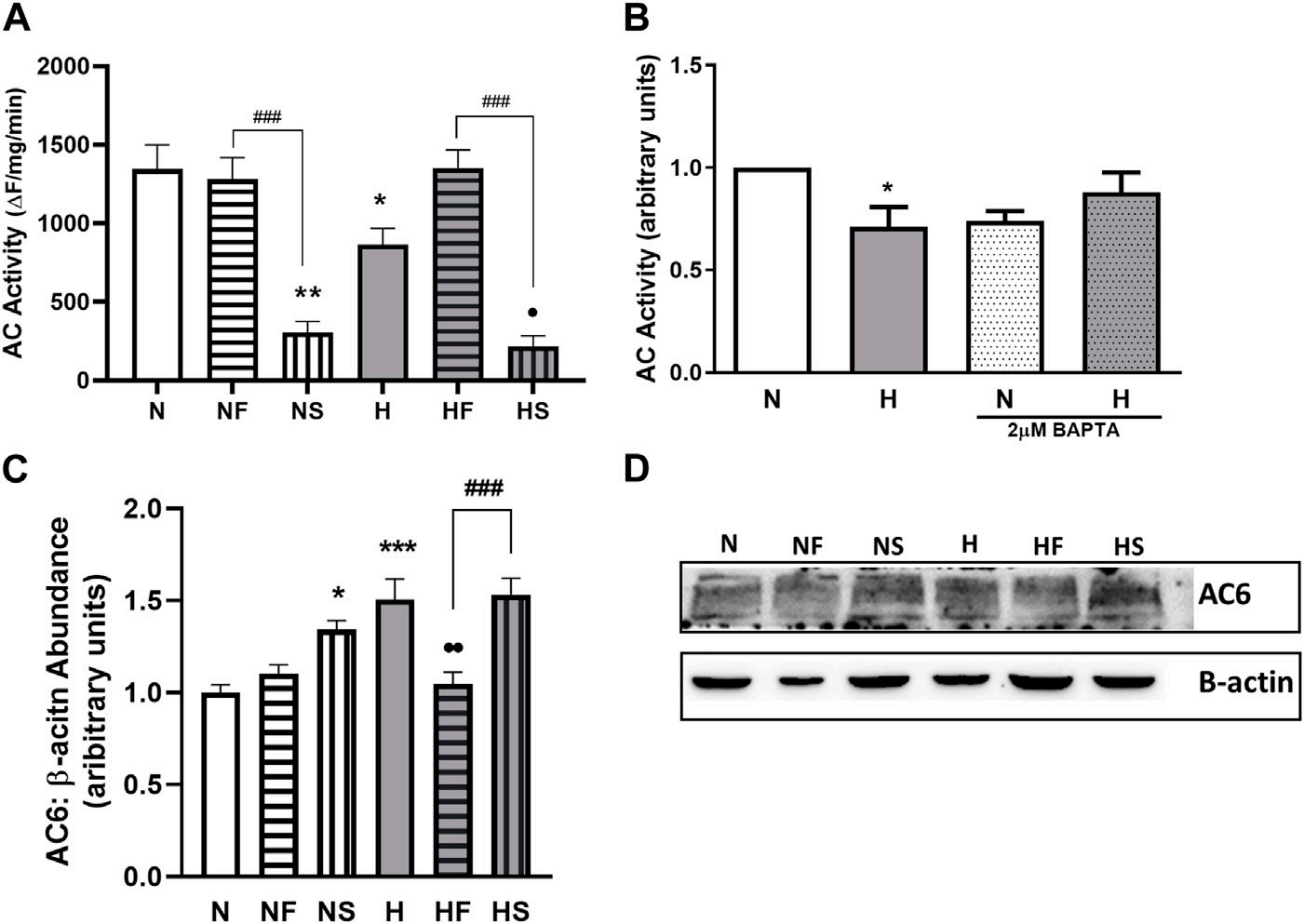
Effect of hypoxia or nitrosylating stress on PASMC relaxation. Adenylyl cyclase (AC) activity was measured in lysates from PASMC exposed to 72 h of normoxia (N; 21%O2) or hypoxia (H; 10%O2) with or without daily addition of 1uM FeTPPS (F) or 1uM SNP (S) **(A)**; N = 5–8, n = 5–14). Intracellular Ca2+ was normalized with chelator BAPTA (2uM, 24 h) prior to lysate collection and AC activity assay **(B)**; N = 2–4, n = 3–4). AC6 abundance was compared by Western blot, normalized to β-actin **(C);** N = 4, n = 4; **(D)**; representative blot shown). *p < 0.05, **p < 0.01, ***p < 0.001compared to N. •*p* < 0.05, • *p* < 0.01 compared to H. ###*p* < 0.001 compared between FeTPPS and SNP treatments within similar O2 environments.

As previously reported, 72 h of hypoxia significantly decreased AC activity (Figure 4A). In addition, treatment of PASMC with SNP impaired AC activity in normoxic and hypoxic PASMC. Addition of FeTPPS to hypoxic PASMC restored AC activity to that of control levels but had no effect on AC activity of normoxic PASMC.

Since Ca2+ can regulate AC activity and we observed elevated basal Ca2+ levels in hypoxic PASMC, we examined whether the impaired AC activity was a result of altered Ca2+ handling by normalizing Ca2+ levels between all samples with the use of the Ca2+ chelator BAPTA. Following Ca2+ chelation, AC activity was similar between control and hypoxic PASMC (Figure 4B).

We next focused on AC6, as our lab has previously shown AC6 to be the most abundant AC isoform expressed in PASMC (Sikarwar et al., 2018), and AC6 expression is known to be driven by hypoxia-inducible factor (HIF) (Simko et al., 2017). Treatment with either SNP (*p* < 0.05) or hypoxia (*p* < 0.001) led to a 50% increase in AC6 abundance (Figures 4C,D), paradoxical to the observed decrease in activity. Treatment of hypoxic PASMC with FeTPPS reduced AC6 abundance back to normoxic control levels (*p* < 0.01).

## Discussion

It was recently recognized that the presence of oxidative and nitrosative stresses complicates the clinical management of PPHN (Rawat et al., 2022). Many of the precipitating factors for PPHN, from prenatal hypoxia to placental insufficiency and intrauterine growth restriction, are themselves sources of fetal oxidative stress; as are treatment modalities frequently used in the neonatal period, such as oxygen supplementation and mechanical ventilation (Perez et al., 2019). ROS and RNS can be measured separately, but are linked though their combination to generate reactive intermediates, prominently peroxynitrite which can directly or indirectly oxidize lipids, proteins and other signaling moieties (Pacher et al., 2007). However, specific mechanisms by which RNS may impair PPHN treatment are generally extrapolated from whole animal ROS/RNS measurements, but not clearly described at a cellular level (Rawat et al., 2022). In this study, we examined the effects of exogenous nitric oxide, as a source of nitrosative stress, on the regulation of contractile and relaxant pathways in neonatal PASMC exposed to hypoxia *in vitro*, as a model of the PPHN pulmonary arterial environment. We analyzed the redox milieu of the hypoxic PASMC, including mitochondrial function as a potential source of oxidative stress, in order to narrow down the sources of nitrosative species following a hypoxic challenge ± nitric oxide addition. We studied intracellular Ca^2+^ mobilization response to a thromboxane challenge as a defined proxy for smooth muscle contraction, and examined the AC-cAMP relaxant pathway as this is known to be activated by regulatory prostanoids and serves as a brake on thromboxane-mediated contraction.

The 72-h hypoxia PPHN model was first established *in vivo* by Haworth (Haworth and Hislop, 1982), and pharmacological changes in this model characterized by Fike (Fike et al., 2003). We have published extensively with this model of 72-h hypoxia in serum-starved PASMC. We use environmental hypoxia to model PPHN PASMC *in vitro*, replicating the *in vivo* PPHN pattern of contractile receptor hypersensitivity and hyperreactivity (Hinton et al., 2006; Hinton et al., 2007; Santhosh et al., 2011; Fediuk et al., 2015), mitochondrial function (Saini-Chohan et al., 2011) and ROS generation (Gong et al., 2010; Awad et al., 2014). Importantly, oxygen concentrations measured in media at the cell growth interface of smooth muscle cells grown in static culture in a normoxic (21% O2) incubator are comparable to the physiological oxygen content in resting tissues (Carreau et al., 2011), while cell growth in a 10% O_2_ incubator halves that oxygen content, into a hypoxic tissue range (Hinton et al., 2019). This gradient owes to the diffusion of oxygen from the gas phase into the vertical column of culture media, in the absence of hemoglobin (Kagawa et al., 2015); and is the basis for *in vitro* hypoxic experimentation (Kagawa et al., 2016).

Hypoxia caused a predictable increase in total ROS in PASMC, as quantified by DCF staining; and a very modest increase in superoxide, identified by DHE staining. These increases were mitigated by addition of the NO donor SNP. NO may be acting in this context as an antioxidant through its rapid interaction with oxidative species such as superoxide (Rawat et al., 2022). Addition of SNP increased intracellular concentration of nitrate and total protein nitrosylation in cell lysates of PASMC. This can be a bit of a red herring, as nitrosylation of different proteins may have markedly differing functional effects (Bhatia et al., 2021) but did verify the overall heightened RNS environment following SNP addition to PASMC. The addition of NO, when combined with superoxide in appropriate quantity and proximity, can give rise to peroxynitrite, which acts as a source of nitroso ions, resulting in protein nitration or nitrosylation (Espey et al., 2002; Bhatia et al., 2021). Hypoxia can favour generation of superoxide (Gong et al., 2010). Effects of NO and of peroxynitrite on systemic arteries are known to differ (Walia et al., 2003). We therefore sought to infer whether the effects of SNP on hypoxic PASMC resulted in conversion of NO plus superoxide to peroxynitrite, by asking whether peroxynitrite can directly act to increment contractile signaling, through use of FeTPPS, a decomposition catalyst which isomerizes peroxynitrite to less reactive nitrate.

Effects of hypoxia on PASMC mitochondrial function were studied to understand intracellular sources of ROS. We found the degree of hypoxia to which PASMC were subjected to in this study decreased basal and maximal mitochondrial activity, while having no effect on mitochondrial morphology or localization. This distinguishes the moderate hypoxia under study, from that in idiopathic pulmonary hypertension which featured visible mitochondrial fragmentation and Warburg metabolism (Ryan et al., 2015). Altered mitochondrial morphology, fission and respiratory suppression (anerobic metabolic shift) are indeed reported in hypoxic PASMC, but following exposure to 1% O_2_ (Parra et al., 2017) rather than the 10% environment utilized here. We previously reported impaired mitochondrial SOD activity in PASMC due to inhibitory nitration of the enzyme following a 10% oxygen exposure (Gong et al., 2010). While this can contribute to superoxide accumulation, the decreased respiratory activity observed at this level of hypoxia may render that accumulation modest.

We focused next on the pathways downstream of prostanoid receptors, as hypoxia increases Gαq coupling of the thromboxane receptor (Fediuk et al., 2012), and may desensitize ligand binding ability of the prostacyclin receptor (Santhosh et al., 2011). We (Hinton et al., 2006; Hinton et al., 2007) and others (Fike et al., 2002; Jankov et al., 2002) have shown that development of thromboxane-mediated arterial constriction is etiologic in pulmonary hypertension due to neonatal hypoxia or oxidative stress in newborns. Hypoxia increases the thromboxane:prostacyclin ratio, increasing pulmonary arterial tone (Fike et al., 2003). The vasodilator prostacyclin is cardioprotective in ischemia-reperfusion injury, while loss of prostacyclin receptor signalling *via* Gαs and AC precipitates spontaneous hypertension (Saha et al., 2008) and hypoxic cardiac dysfunction (Rohlicek et al., 2005). Decreased basal and agonist-mediated cAMP was reported by our group (Santhosh et al., 2011; Sikarwar et al., 2018) and others (Gao and Raj, 2010; Sylvester et al., 2012) studying the acutely hypoxic pulmonary artery. In the current study, addition of a NO donor appeared to normalize the increased baseline Ca^2+^ in hypoxic PASMC. Peroxynitrite scavenging also had this effect, suggesting the elevation of basal Ca^2+^ may be a ROS-mediated phenomenon independent of receptors. Potassium ion channels, well known to be oxygen sensors (Michelakis et al., 1995), regulate resting Ca^2+^ in PASMC. However, thromboxane-mediated Ca^2+^ mobilization was higher in hypoxic PASMC treated with SNP, while FeTPPS reverted the elevated Ca^2+^ response of hypoxic PASMC to that of normoxic cells. Concurrent treatment of hypoxic PASMC with SNP and FeTPPS ablated the SNP-mediated increase in Ca^2+^ response to a thromboxane challenge, suggesting the direct involvement of peroxynitrite in this mechanism. Hypoxia itself increases smooth muscle thromboxane sensitivity by more than one method. It decreases serine phosphorylation of the thromboxane receptor resulting in agonist hyperresponsiveness (Santhosh et al., 2014), while also increasing palmitoylation of the thromboxane receptor’s cognate G protein Gαq increasing its association with the thromboxane receptor and augmenting the IP_3_ signal for Ca^2+^ release (Sikarwar et al., 2014). We observed no change in abundance of the typical contractile phenotype markers in treated PASMC, nor any change in abundance of phospholipase C which generates IP_3_. We have previously reported decreased cell surface expression of thromboxane receptor in PASMC following hypoxia exposure, associated with increased receptor internalization (Hinton et al., 2007; Fediuk et al., 2015); the increased Ca^2+^ mobilization is therefore not driven by increased receptor abundance. We speculate that RNS-mediated changes in contractile pathway regulation may include post-translational modifications of the receptor G protein complex, augmenting signals for calcium mobilization. Gαi association with membrane receptors is known to increase when it is nitrosylated (Chao et al., 2021). We also reported in PASMC a switch from Gαs-to Gαi-signalling in a nitrosylating environment (Bhagirath et al., 2022). Receptor G protein coupling dynamics deserve further examination in presence of ROS/RNS in order to elucidate the mechanisms for NO augmentation of hypoxic Ca^2+^ mobilization.

On the relaxant side, AC activity was examined independent of receptor activity, using a terbium norfloxacin reporter sensitive to AC activity. Loss of lanthanide-quinolone luminescence due to catalysis of lanthanide-bound ATP by AC is an indirect but reasonably specific indicator of ATP turnover to cAMP, as the bound ATP is not available for catalysis by other ATPases which catalyze only free ATP (Spangler et al., 2008). By utilizing this method both protein content and substrate concentration can be controlled and AC activity determined independent of G protein activation. We previously reported that hypoxia inhibits AC activity *via* AC protein nitrosylation (Sikarwar et al., 2018). Given the difference in Ca^2+^ baseline between normoxic and hypoxic PASMC, we first sought to control for Ca2+-mediated inhibition of AC activity. Chelation of Ca^2+^ did increase hypoxic AC activity to a degree. In addition, treatment with FeTPPS also increased AC activity in hypoxic cells. But upon addition of SNP, a marked inhibition of AC activity was observed in both normoxic and hypoxic PASMC. Paradoxically, this is associated with increased abundance of AC, in particular of the predominant AC6 isoform; this is understandable given the presence of a HIF-response element in AC6 promoter (Simko et al., 2017). Certain AC isoforms, including AC6, have been identified as sensitive to catalytic inhibition by NO (McVey et al., 1999; Goldstein et al., 2002); our data adds functional context in pulmonary artery PASMC. This finding also mirrors the mechanism of inhibition of the other vascular nucleotide cyclase, guanylate cyclase, which is known to occur during inhaled NO therapy (Gladwin, 2006) and during treatment with nitrates (Sayed et al., 2008). In that case, nitrosylation of cysteines at the catalytic site inhibits GTP catalysis, causing NO resistance (Kokkola et al., 2005; Evgenov et al., 2006). Thus, NO-mediated inhibition of AC during PPHN treatment could result in attenuation of all relaxant pathways, resulting in unchecked contractile signaling.

In summary, our study represents a primary survey of the effects of NO on the generation of major second messengers in neonatal pulmonary artery PASMC. The mechanisms for these effects will require further investigation. Limitations of this study include the NO donor used. As a lipophilic gas, NO can freely diffuse across membranes up to 100 μm (Lancaster, 1997). Endogenous tissue NO concentrations can range 10–100 nM with a half-life of 3–5 s (Czapski and Goldstein, 1995). It is difficult to ascertain tissue concentrations of NO following inhaled therapy. Kinetics of NO release varies between different NO donors, and can be affected by light or by media CO2 concentration which will influence NO steady state (He and Frost, 2016). NO released from SNP reaches a sustained steady state in physiological range by 30 min after addition, without NO dumping (Bradley and Steinert, 2015). We confirmed unchanged cell viability after use of this agent, as iron and cyanide release are reported from SNP. This iron can also induce hemoxygenase and increase cAMP generation (Kim et al., 2006), although we observed the opposite effect of SNP on cAMP. While correlation of media NO after SNP addition with tissue NO after inhaled NO administration is not directly possible, we feel this is a reasonable *in vitro* model to determine the effects of NO on smooth muscle physiological pathways. The current study is limited to myocytes obtained in first passage from normal neonatal pulmonary arteries and exposed to hypoxia or other treatments *in vitro*; we focused on second messenger generation, which is best dissected using cultured myocytes rather than in whole tissues where the bioavailability of activators or inhibitors is sometimes limited by tissue penetration. However, we previously reported close correlation of the measured second messengers with myographic functional responses of whole vessels from PPHN animals (Fediuk et al., 2014; Sikarwar et al., 2018), and therefore posit that the data presented may have implications for smooth muscle behaviour in PPHN pulmonary artery. Further *ex-vivo* myographic study of NO effects on the cumulative contractile responses of PPHN arteries is warranted, employing inhibition of opposing cGMP-mediated pathways to discern these off-target NO effects.

In conclusion, NO independently augments contraction and inhibits relaxation pathways in hypoxic PASMC. This is temporally linked to the formation of nitrogen radicals and may involve increased protein nitrosylation. Together, these phenomena may impede the effectiveness of NO in treating hypoxic pulmonary hypertension.

## Data Availability

The original contributions presented in the study are included in the article/Supplementary Material, further inquiries can be directed to the corresponding author.

## References

[B1] Awad H. Nolette N. Hinton M. Dakshinamurti S. (2014). AMPK and FoxO1 regulate catalase expression in hypoxic pulmonary arterial smooth muscle. Pediatr. Pulmonol. 49 (9), 885–897. 10.1002/ppul.22919 24167160

[B2] Barrington K. J. Finer N. Pennaforte T. Altit G. (2017). Nitric oxide for respiratory failure in infants born at or near term. Cochrane Database Syst. Rev. 1, CD000399. 10.1002/14651858.CD000399.pub3 28056166 PMC6464941

[B3] Bhagirath A. Y. Bhatia V. Medapati M. R. Singh N. Hinton M. Chelikani P. (2022). Critical cysteines in the functional interaction of adenylyl cyclase isoform 6 with Gαs. FASEB BioAdvances 4 (3), 180–196. 10.1096/fba.2021-00073 35664968 PMC9159366

[B4] Bhatia V. Elnagary L. Dakshinamurti S. (2021). Tracing the path of inhaled nitric oxide: Biological consequences of protein nitrosylation. Pediatr. Pulmonol. 56 (2), 525–538. 10.1002/ppul.25201 33289321

[B5] Bhutani V. K. (2008). Developing a systems approach to prevent meconium aspiration syndrome: Lessons learned from multinational studies. J. Perinatol. 28 (3), S30–S35. 10.1038/jp.2008.159 19057608

[B6] Bradley S. A. Steinert J. R. (2015). Characterisation and comparison of temporal release profiles of nitric oxide generating donors. J. Neurosci. Methods 245, 116–124. 10.1016/j.jneumeth.2015.02.024 25749567 PMC4401449

[B7] Carreau A. El Hafny-Rahbi B. Matejuk A. Grillon C. Kieda C. (2011). Why is the partial oxygen pressure of human tissues a crucial parameter? Small molecules and hypoxia. J. Cell Mol. Med. 15 (6), 1239–1253. 10.1111/j.1582-4934.2011.01258.x 21251211 PMC4373326

[B8] Chao M. L. Luo S. Zhang C. Zhou X. Zhou M. Wang J. (2021). S-nitrosylation-mediated coupling of G-protein alpha-2 with CXCR5 induces Hippo/YAP-dependent diabetes-accelerated atherosclerosis. Nat. Commun. 12 (1), 4452. 10.1038/s41467-021-24736-y 34294713 PMC8298471

[B9] Clark R. H. Kueser T. J. Walker M. W. Southgate W. M. Huckaby J. L. Perez J. A. (2000). Low-dose nitric oxide therapy for persistent pulmonary hypertension of the newborn. Clinical Inhaled Nitric Oxide Research Group. N. Engl. J. Med. 342 (7), 469–474. 10.1056/NEJM200002173420704 10675427

[B10] Czapski G. Goldstein S. (1995). The role of the reactions of.NO with superoxide and oxygen in biological systems: A kinetic approach. Free Radic. Biol. Med. 19 (6), 785–794. 10.1016/0891-5849(95)00081-8 8582651

[B11] Dakshinamurti S. (2005). Pathophysiologic mechanisms of persistent pulmonary hypertension of the newborn. Pediatr. Pulmonol. 39 (6), 492–503. 10.1002/ppul.20201 15789439

[B12] Espey M. G. Thomas D. D. Miranda K. M. Wink D. A. (2002). Focusing of nitric oxide mediated nitrosation and oxidative nitrosylation as a consequence of reaction with superoxide. Proc. Natl. Acad. Sci. U. S. A. 99 (17), 11127–11132. 10.1073/pnas.152157599 12177414 PMC123221

[B13] Evgenov O. V. Pacher P. Schmidt P. M. Hasko G. Schmidt H. H. Stasch J. P. (2006). NO-Independent stimulators and activators of soluble guanylate cyclase: Discovery and therapeutic potential. Nat. Rev. Drug Discov. 5 (9), 755–768. 10.1038/nrd2038 16955067 PMC2225477

[B14] Farrow K. N. Fliman P. Steinhorn R. H. (2005). The diseases treated with ECMO: Focus on PPHN. Semin. Perinatol. 29 (1), 8–14. 10.1053/j.semperi.2005.02.003 15921147

[B15] Fediuk J. Gutsol A. Nolette N. Dakshinamurti S. (2012). Thromboxane-induced actin polymerization in hypoxic pulmonary artery is independent of Rho. Am. J. Physiol. Lung Cell Mol. Physiol. 302 (1), L13–L26. 10.1152/ajplung.00016.2011 21926266

[B16] Fediuk J. Sikarwar A. S. Lizotte P. P. Hinton M. Nolette N. Dakshinamurti S. (2015). Hypoxia increases pulmonary arterial thromboxane receptor internalization independent of receptor sensitization. Pulm. Pharmacol. Ther. 30, 1–10. 10.1016/j.pupt.2014.10.001 25312900

[B17] Fediuk J. Sikarwar A. S. Nolette N. Dakshinamurti S. (2014). Thromboxane-induced actin polymerization in hypoxic neonatal pulmonary arterial myocytes involves Cdc42 signaling. Am. J. Physiol. Lung Cell Mol. Physiol. 307 (11), L877–L887. 10.1152/ajplung.00036.2014 25281640

[B18] Fike C. D. Kaplowitz M. R. Pfister S. L. (2003). Arachidonic acid metabolites and an early stage of pulmonary hypertension in chronically hypoxic newborn pigs. Am. J. Physiol. Lung Cell Mol. Physiol. 284 (2), L316–L323. 10.1152/ajplung.00228.2002 12388340

[B19] Fike C. D. Pfister S. L. Kaplowitz M. R. Madden J. A. (2002). Cyclooxygenase contracting factors and altered pulmonary vascular responses in chronically hypoxic newborn pigs. J. Appl. Physiol. 92 (1), 67–74. 10.1152/jappl.2002.92.1.67 11744644

[B20] Finer N. N. Barrington K. J. (2006). Nitric oxide for respiratory failure in infants born at or near term. Cochrane Database Syst. Rev. 4, CD000399. 10.1002/14651858.CD000399.pub2 17054129

[B21] Gao Y. Raj J. U. (2010). Regulation of the pulmonary circulation in the fetus and newborn. Physiol. Rev. 90(4), 1291–1335. 10.1152/physrev.00032.2009 20959617

[B22] Gladwin M. T. (2006). Deconstructing endothelial dysfunction: Soluble guanylyl cyclase oxidation and the NO resistance syndrome. J. Clin. Invest. 116 (9), 2330–2332. 10.1172/JCI29807 16955136 PMC1555666

[B23] Goldman A. P. Tasker R. C. Haworth S. G. Sigston P. E. Macrae D. J. (1996). Four patterns of response to inhaled nitric oxide for persistent pulmonary hypertension of the newborn. Pediatrics 98 (1), 706–713. 10.1542/peds.98.4.706 8885950

[B24] Goldstein J. Silberstein C. Ibarra C. (2002). Adenylyl cyclase types I and VI but not II and V are selectively inhibited by nitric oxide. Braz J. Med. Biol. Res. 35 (2), 145–151. 10.1590/s0100-879x2002000200002 11847517

[B25] Gong Y. Yi M. Fediuk J. Lizotte P. P. Dakshinamurti S. (2010). Hypoxic neonatal pulmonary arterial myocytes are sensitized to ROS-generated 8-isoprostane. Free Radic. Biol. Med. 48 (7), 882–894. 10.1016/j.freeradbiomed.2010.01.009 20079425

[B26] Gould N. Doulias P. T. Tenopoulou M. Raju K. Ischiropoulos H. (2013). Regulation of protein function and signaling by reversible cysteine S-nitrosylation. J. Biol. Chem. 288 (37), 26473–26479. 10.1074/jbc.R113.460261 23861393 PMC3772194

[B27] Grynkiewicz G. Poenie M. Tsien R. Y. (1985). A new generation of Ca2+ indicators with greatly improved fluorescence properties. J. Biol. Chem. 260 (6), 3440–3450. 10.1016/s0021-9258(19)83641-4 3838314

[B28] Halayko A. J. Rector E. Stephens N. L. (1997). Characterization of molecular determinants of smooth muscle cell heterogeneity. Can. J. Physiol. Pharmacol. 75 (7), 917–929. 10.1139/cjpp-75-7-917 9315361

[B29] Haworth S. G. Hislop A. A. (1982). Effect of hypoxia on adaptation of the pulmonary circulation to extra-uterine life in the pig. Cardiovasc Res. 16 (6), 293–303. 10.1093/cvr/16.6.293 7105097

[B30] He W. Frost M. C. (2016). Direct measurement of actual levels of nitric oxide (NO) in cell culture conditions using soluble NO donors. Redox Biol. 9, 1–14. 10.1016/j.redox.2016.05.002 27236086 PMC4899081

[B31] Hinton M. Gutsol A. Dakshinamurti S. (2007). Thromboxane hypersensitivity in hypoxic pulmonary artery myocytes: Altered TP receptor localization and kinetics. Am. J. Physiol. Lung Cell Mol. Physiol. 292 (3), L654–L663. 10.1152/ajplung.00229.2006 17085527

[B32] Hinton M. Mellow L. Halayko A. J. Gutsol A. Dakshinamurti S. (2006). Hypoxia induces hypersensitivity and hyperreactivity to thromboxane receptor agonist in neonatal pulmonary arterial myocytes. Am. J. Physiol. Lung Cell Mol. Physiol. 290 (2), L375–L384. 10.1152/ajplung.00307.2005 16214814

[B33] Hinton M. Sikarwar A. S. Dakshinamurti S. (2019). Preparation of pulmonary artery myocytes and rings to study vasoactive GPCRs. Methods Mol. Biol. 1947, 389–401. 10.1007/978-1-4939-9121-1_23 30969430

[B34] Jankov R. P. Belcastro R. Ovcina E. Lee J. Massaeli H. Lye S. J. (2002). Thromboxane A(2) receptors mediate pulmonary hypertension in 60% oxygen-exposed newborn rats by a cyclooxygenase-independent mechanism. Am. J. Respir. Crit. Care Med. 166 (2), 208–214. 10.1164/rccm.200112-124OC 12119234

[B35] Kagawa Y. Matsuura K. Shimizu T. Tsuneda S. (2015). Direct measurement of local dissolved oxygen concentration spatial profiles in a cell culture environment. Biotechnol. Bioeng. 112 (6), 1263–1274. 10.1002/bit.25531 25565074

[B36] Kagawa Y. Miyahara H. Ota Y. Tsuneda S. (2016). System for measuring oxygen consumption rates of mammalian cells in static culture under hypoxic conditions. Biotechnol. Prog. 32 (1), 189–197. 10.1002/btpr.2202 26558344

[B37] Kim H. J. Tsoy I. Park M. K. Lee Y. S. Lee J. H. Seo H. G. (2006). Iron released by sodium nitroprusside contributes to heme oxygenase-1 induction via the cAMP-protein kinase A-mitogen-activated protein kinase pathway in RAW 264.7 cells. Mol. Pharmacol. 69 (5), 1633–1640. 10.1124/mol.105.020487 16439612

[B38] Kokkola T. Savinainen J. R. Monkkonen K. S. Retamal M. D. Laitinen J. T. (2005). S-nitrosothiols modulate G protein-coupled receptor signaling in a reversible and highly receptor-specific manner. BMC Cell Biol. 6 (1), 21. 10.1186/1471-2121-6-21 15850493 PMC1090567

[B39] Lakshminrusimha S. Russell J. A. Wedgwood S. Gugino S. F. Kazzaz J. A. Davis J. M. (2006). Superoxide dismutase improves oxygenation and reduces oxidation in neonatal pulmonary hypertension. Am. J. Respir. Crit. care Med. 174 (12), 1370–1377. 10.1164/rccm.200605-676OC 17008638 PMC2111046

[B40] Lancaster J. R. Jr. (1997). A tutorial on the diffusibility and reactivity of free nitric oxide. Nitric Oxide 1 (1), 18–30. 10.1006/niox.1996.0112 9701041

[B41] McVey M. Hill J. Howlett A. Klein C. (1999). Adenylyl cyclase, a coincidence detector for nitric oxide. J. Biol. Chem. 274 (27), 18887–18892. 10.1074/jbc.274.27.18887 10383385

[B42] Michelakis E. D. Archer S. L. Weir E. K. (1995). Acute hypoxic pulmonary vasoconstriction: A model of oxygen sensing. Physiol. Res. 44 (6), 361–367. 8798271

[B43] Pacher P. Beckman J. S. Liaudet L. (2007). Nitric oxide and peroxynitrite in health and disease. Physiol. Rev. 87 (1), 315–424. 10.1152/physrev.00029.2006 17237348 PMC2248324

[B44] Parra V. Bravo-Sagua R. Norambuena-Soto I. Hernandez-Fuentes C. P. Gomez-Contreras A. G. Verdejo H. E. (2017). Inhibition of mitochondrial fission prevents hypoxia-induced metabolic shift and cellular proliferation of pulmonary arterial smooth muscle cells. Biochim. Biophys. Acta Mol. Basis Dis. 1863 (11), 2891–2903. 10.1016/j.bbadis.2017.07.018 28739174

[B45] Pedersen J. Hedegaard E. R. Simonsen U. Kruger M. Infanger M. Grimm D. (2018). Current and future treatments for persistent pulmonary hypertension in the newborn. Basic Clin. Pharmacol. Toxicol. 123 (4), 392–406. 10.1111/bcpt.13051 29855164

[B46] Perez M. Robbins M. E. Revhaug C. Saugstad O. D. (2019). Oxygen radical disease in the newborn, revisited: Oxidative stress and disease in the newborn period. Free Radic. Biol. Med. 142, 61–72. 10.1016/j.freeradbiomed.2019.03.035 30954546 PMC6791125

[B47] Qin Y. Dey A. Daaka Y. (2013). Protein s-nitrosylation measurement. Methods Enzymol. 522, 409–425. 10.1016/B978-0-12-407865-9.00019-4 23374195

[B48] Rawat M. Lakshminrusimha S. Vento M. (2022). Pulmonary hypertension and oxidative stress: Where is the link? Semin. Fetal Neonatal Med. 27 (4), 101347. 10.1016/j.siny.2022.101347 35473693 PMC11151383

[B49] Rohlicek C. V. Viau S. Trieu P. Hebert T. E. (2005). Effects of neonatal hypoxia in the rat on inotropic stimulation of the adult heart. Cardiovasc Res. 65 (4), 861–868. 10.1016/j.cardiores.2004.12.003 15721866

[B50] Ryan J. Dasgupta A. Huston J. Chen K. H. Archer S. L. (2015). Mitochondrial dynamics in pulmonary arterial hypertension. J. Mol. Med. Berl. 93 (3), 229–242. 10.1007/s00109-015-1263-5 25672499 PMC4339102

[B51] Saha S. Li Y. Anand-Srivastava M. B. (2008). Reduced levels of cyclic AMP contribute to the enhanced oxidative stress in vascular smooth muscle cells from spontaneously hypertensive rats. Can. J. Physiol. Pharmacol. 86 (4), 190–198. 10.1139/Y08-012 18418428

[B52] Saini-Chohan H. K. Dakshinamurti S. Taylor W. A. Shen G. X. Murphy R. Sparagna G. C. (2011). Persistent pulmonary hypertension results in reduced tetralinoleoyl-cardiolipin and mitochondrial complex II + III during the development of right ventricular hypertrophy in the neonatal pig heart. Am. J. Physiol. Heart Circ. Physiol. 301 (4), H1415–H1424. 10.1152/ajpheart.00247.2011 21841017

[B53] Santhosh K. T. Elkhateeb O. Nolette N. Outbih O. Halayko A. J. Dakshinamurti S. (2011). Milrinone attenuates thromboxane receptor-mediated hyperresponsiveness in hypoxic pulmonary arterial myocytes. Br. J. Pharmacol. 163 (6), 1223–1236. 10.1111/j.1476-5381.2011.01306.x 21385177 PMC3144536

[B54] Santhosh K. T. Sikarwar A. S. Hinton M. Chelikani P. Dakshinamurti S. (2014). Thromboxane receptor hyper-responsiveness in hypoxic pulmonary hypertension requires serine 324. Br. J. Pharmacol. 171 (3), 676–687. 10.1111/bph.12487 24490858 PMC3969080

[B55] Sayed N. Kim D. D. Fioramonti X. Iwahashi T. Duran W. N. Beuve A. (2008). Nitroglycerin-induced S-nitrosylation and desensitization of soluble guanylyl cyclase contribute to nitrate tolerance. Circ. Res. 103 (6), 606–614. 10.1161/CIRCRESAHA.108.175133 18669924 PMC2737267

[B56] Shimoda L. A. Sham J. S. Shimoda T. H. Sylvester J. T. (2000). L-type Ca(2+) channels, resting [Ca(2+)](i), and ET-1-induced responses in chronically hypoxic pulmonary myocytes. Am. J. Physiol. Lung Cell Mol. Physiol. 279 (5), L884–L894. 10.1152/ajplung.2000.279.5.L884 11053024

[B57] Sikarwar A. S. Hinton M. Santhosh K. T. Chelikani P. Dakshinamurti S. (2014). Palmitoylation of Gαq determines its association with the thromboxane receptor in hypoxic pulmonary hypertension. Am. J. Respir. Cell Mol. Biol. 50 (1), 135–143. 10.1165/rcmb.2013-0085OC 23962128

[B58] Sikarwar A. S. Hinton M. Santhosh K. T. Dhanaraj P. Talabis M. Chelikani P. (2018). Hypoxia inhibits adenylyl cyclase catalytic activity in a porcine model of persistent pulmonary hypertension of the newborn. Am. J. Physiol. Lung Cell Mol. Physiol. 315 (6), L933–L944. 10.1152/ajplung.00130.2018 30234376

[B59] Simko V. Iuliano F. Sevcikova A. Labudova M. Barathova M. Radvak P. (2017). Hypoxia induces cancer-associated cAMP/PKA signalling through HIF-mediated transcriptional control of adenylyl cyclases VI and VII. Sci. Rep. 7 (1), 10121. 10.1038/s41598-017-09549-8 28860539 PMC5578998

[B60] Spangler C. M. Spangler C. Gottle M. Shen Y. Tang W. J. Seifert R. (2008). A fluorimetric assay for real-time monitoring of adenylyl cyclase activity based on terbium norfloxacin. Anal. Biochem. 381 (1), 86–93. 10.1016/j.ab.2008.06.014 18601890

[B61] Steinhorn R. H. Albert G. Swartz D. D. Russell J. A. Levine C. R. Davis J. M. (2001). Recombinant human superoxide dismutase enhances the effect of inhaled nitric oxide in persistent pulmonary hypertension. Am. J. Respir. Crit. Care Med. 164 (5), 834–839. 10.1164/ajrccm.164.5.2010104 11549542

[B62] Steurer M. A. Jelliffe-Pawlowski L. L. Baer R. J. Partridge J. C. Rogers E. E. Keller R. L. (2017). Persistent pulmonary hypertension of the newborn in late preterm and term infants in California. Pediatrics 139 (1), e20161165. 10.1542/peds.2016-1165 27940508

[B63] Sylvester J. T. Shimoda L. A. Aaronson P. I. Ward J. P. (2012). Hypoxic pulmonary vasoconstriction. Physiol. Rev. 92 (1), 367–520. 10.1152/physrev.00041.2010 22298659 PMC9469196

[B64] Walia M. Samson S. E. Schmidt T. Best K. Whittington M. Kwan C. Y. (2003). Peroxynitrite and nitric oxide differ in their effects on pig coronary artery smooth muscle. Am. J. physiology.Cell physiology 284 (3), C649–C657. 10.1152/ajpcell.00405.2002 12431912

